# Sparse-Aware Bias-Compensated Adaptive Filtering Algorithms Using the Maximum Correntropy Criterion for Sparse System Identification with Noisy Input

**DOI:** 10.3390/e20060407

**Published:** 2018-05-25

**Authors:** Wentao Ma, Dongqiao Zheng, Zhiyu Zhang, Jiandong Duan, Jinzhe Qiu, Xianzhi Hu

**Affiliations:** 1 School of Automation and Information Engineering, Xi’an University of Technology, Xi’an 710048, China; 2 State Key Laboratory of Electrical Insulation and Power Equipment, Xi’an Jiaotong University, Xi’an 710049, China; 3 Management Center of Internet Information, Xi’an University of Technology, Xi’an 710048, China

**Keywords:** bias-compensated, correntropy-induced metric, maximum correntropy criterion, noisy input, proportionate update, sparse system identification

## Abstract

To address the sparse system identification problem under noisy input and non-Gaussian output measurement noise, two novel types of sparse bias-compensated normalized maximum correntropy criterion algorithms are developed, which are capable of eliminating the impact of non-Gaussian measurement noise and noisy input. The first is developed by using the correntropy-induced metric as the sparsity penalty constraint, which is a smoothed approximation of the $\ell_{0}$ norm. The second is designed using the proportionate update scheme, which facilitates the close tracking of system parameter change. Simulation results confirm that the proposed algorithms can effectively improve the identification performance compared with other algorithms presented in the literature for the sparse system identification problem.

## 1. Introduction

The least mean square (LMS), normalized least mean square (NLMS), least mean fourth (LMF), and normalized least mean fourth (NLMF) algorithms have been widely used in adaptive system identification, channel estimation, and echo cancellation [1,2,3,4] due to their low complexity and easy implementation. However, their performance is usually degraded severely when they are subject to output noise with non-Gaussian characteristics. Correspondingly, many robust adaptive filter algorithms (AFAs) have been developed to reduce the impact of non-Gaussian measurement noise, such as the maximum correntropy criterion (MCC) [5,6,7,8], least mean mixed norm (LMMN) [9], and least mean absolute deviation (LMAD) [10] algorithms, and so on. Among them, the MCC has been utilized to design different robust algorithms (including the diffusion MCC [11], kernel MCC [12], generalized MCC [13], group-constrained MCC, and reweight group-constrained MCC [14] algorithms, and so on [15,16]) to solve different engineering problems.

Although those algorithms mentioned above show robustness against non-Gaussian measurement noise, they show weak performance for sparse system identification (SSI) problems because they do not take advantage of prior knowledge of the system. As a result, two main technologies have been developed to address the SSI problem: one using the compressed sensing approach [17,18], and the other employing the proportionate update scheme [19]. At present, the zero attracting (ZA) algorithm and reweight zero attracting algorithm belonging to the former (such as ZANLMS [20], ZANLMF [21], ZAMCC [22] and General ZA-Proportionate Normalized MCC [23]) have been proposed based on the sparse penalty term (SPE). The correntropy-induced metric (CIM) [5], as an effective SPE, has been utilized to improve the performance of the algorithm in SSI, resulting in the CIM-NLMS, CIM-NLMF, CIM-LMMN, and CIM-MCC algorithms [22,24,25]. Correspondingly, the latter algorithms are proportionate-type AFAs (including proportionate NLMS [19], proportionate NLMF [26], proportionate MCC [27], and so on) which use the gain matrix to improve performance. Although those algorithms above make full use of the sparsity of the system, they lack consideration of the noisy input problem. As a result, more and more bias-compensated aware AFAs with the unbiasedness criterion have been developed to eliminate the influence from noisy input signals [28,29,30,31]. These include, for example, the bias-compensated NLMS (BCNLMS) algorithm [28,29,30], bias-compensated NLMF algorithm [31], bias-compensated affine projection algorithm [32], bias-compensated NMCC (BCNMCC) [33], and so on. However, they do not consider the sparsity of the system.

On the basis of the analysis above, we develop two novel algorithms called bias-compensated NMCC with CIM penalty (CIM-BCNMCC) and bias-compensated proportionate NMCC (BCPNMCC) in this work. The former introduces the CIM into the BCNMCC algorithm, while the latter combines the unbiasedness criterion and the PNMCC algorithm. Both of them can achieve better performance than MCC, CIM-MCC, and BCNMCC for SSI under noisy input and non-Gaussian measurement noise.

The rest of the paper is organized as follows: In Section 2, the BCNMCC algorithm is briefly reviewed. In Section 3, the CIM-BCNMCC and BCPNMCC algorithms are developed. In Section 4, simulation results are presented to evaluate the performance of the proposed algorithms. Finally, conclusions are made in Section 5.

## 2. Review of the BCNMCC

### 2.1. NMCC

Consider an FIR model with a sample of the observed output signal d(n) defined as

(1) $$d(n)=\;u{\left(n\right)}^{T}w^{o}+v(n)$$

where $u(n)={\left[u(n),u(n-1),\ldots ,u(n-M+1)\right]}^{T}$ denotes the input signal; $w^{o}={\left[w_{1}^{o},w_{2}^{o},\ldots ,w_{M}^{o}\right]}^{T}$ is the estimated M-taps weight vector; and v(n) denotes the output measurement noise at time index *n*, described as non-Gaussian in this work. In order to eliminate the impact of the impulsive noise, the MCC is usually used as a cost function to design robust AFAs, and it is defined as [5]

(2) $$J_{MCC}(n)=\exp(-\frac{e^{2}(n)}{2\sigma^{2}})$$

where $e(n)=d(n)-u^{T}(n)w(n)$ is the instantaneous estimation error, $w(n)={\left[w_{1}(n),w_{2}(n),\ldots ,w_{M}(n)\right]}^{T}$ denotes the estimated tap coefficients vector of $w^{o}={\left[w_{1}^{o},w_{2}^{o},\ldots ,w_{M}^{o}\right]}^{T}$, and σ represents the kernel width which can be manually set. Using Equation (2) and the gradient method, we have

(3) $$w(n+1)=w(n)+\mu \exp(-\frac{e^{2}(n)}{2\sigma^{2}})\frac{e(n)u(n)}{u^{T}(n)u(n)+\varepsilon }$$

where μ denotes the step size, and ε is a positive parameter. Equation (3) is the update equation of NMCC.

### 2.2. BCNMCC

To identify the system parameter under noisy input and non-Gaussian output measurement noise, a bias-compensated normalized maximum correntropy criterion (BCNCC) algorithm has been previously proposed [33]. The u(n) and e(n) terms should be replaced by $\bar{u}(n)$ and $\bar{e}(n)$ in Equation (3) due to the noisy input, and we define the noisy input vector as

(4) $$\bar{u}(n)=u(n)+v_{in}(n)$$

where $v_{in}(n)={\left[v(n),v(n-1),\ldots ,v(n-M+1)\right]}^{T}$ is the input noise with zero mean and variance $\sigma_{in}^{2}$.

(5) $$\bar{e}(n)=d(n)-{\bar{u}}^{T}(n)w(n)$$

To compensate the bias caused by the input noise, the BCNMCC algorithm is developed by introducing a bias-compensation term B(n) into the weight update equation of the normalized MCC algorithm as follows:

(6) $$w(n+1)=w(n)+\mu \exp(-\frac{{\bar{e}}^{2}(n)}{2\sigma^{2}})\frac{\bar{e}(n)\bar{u}(n)}{{\bar{u}}^{T}(n)\bar{u}(n)+\varepsilon }+B(n).$$

To compute B(n), using the weight–error vector (WEV) $\tilde{w}(n)=w^{o}-w(n)$ and Equation (6) yields

(7) $$\tilde{w}(n+1)=\tilde{w}(n)-\mu \exp(-\frac{{\bar{e}}^{2}(n)}{2\sigma^{2}})\frac{\bar{e}(n)\bar{u}(n)}{{\bar{u}}^{T}(n)\bar{u}(n)+\varepsilon }-B(n).$$

Furthermore, the following unbiasedness criterion [28] is employed

(8) $$\begin{array}{ccc} E[\tilde{w}(n+1)|\bar{u}(n)]=0 & \mathrm{whenever} & E[\tilde{w}(n)|\bar{u}(n)]=0 \end{array}$$

and by some simplified calculations, we have

(9) $$B(n)=\mu \exp(-\frac{v^{2}(n)}{2\sigma^{2}})\frac{\sigma_{in}^{2}}{{\bar{u}}^{T}(n)\bar{u}(n)+\varepsilon }.$$

Now, combining Equations (6) and (9), we obtain

(10) $$w(n+1)=\left(1+\mu \exp(\frac{v^{2}(n)}{-2\sigma^{2}})\frac{\sigma_{in}^{2}}{{\bar{u}}^{T}(n)\bar{u}(n)+\varepsilon }\right)w(n)+\mu \exp(-\frac{{\bar{e}}^{2}(n)}{2\sigma^{2}})\frac{\bar{e}(n)\bar{u}(n)}{{\bar{u}}^{T}(n)\bar{u}(n)+\varepsilon }$$

which is the weight update equation of the BCNMCC algorithm.

## 3. Sparse-Aware BCNMCC Algorithms

### 3.1. CIM-BCNMCC

#### 3.1.1. Correntropy-Induced Metric

In this subsection, we focus on developing a novel sparse BCNMCC algorithm with CIM to solve the SSI problem. Here, we first briefly review the CIM. Given two vectors $X={\left[x_{1},x_{2},\ldots x_{N}\right]}^{T}$ and $Y={\left[y_{1},y_{2},\ldots y_{N}\right]}^{T}$ in the sample space, the CIM is defined as

(11) $$CIM(X,Y)={\left(\kappa (0)-\hat{V}(X,Y)\right)}^{1/2}$$

where $\kappa (0)=1/(\sigma \sqrt{2\pi })$*, and* $\hat{V}(X,Y)=1/N\sum_{i=1}^{N}\kappa (x_{i},y_{i})$ is the sample estimation of the correntropy. The most popular kernel in correntropy is the Gaussian kernel $\kappa (x,y)=\frac{1}{\sigma \sqrt{2\pi }}\exp(-\frac{e^{2}}{2\sigma^{2}})$*,* e=x−y*. The* $\ell_{0}$ norm of the vector $X={\left[x_{1},x_{2},\ldots ,x_{N}\right]}^{T}$ can be approximated by

(12) $$||X_{0}||\sim CIM^{2}(X,0)=\frac{\kappa (0)}{N}\sum_{i=1}^{N}\left(1-\exp(-x_{i}^{2}/2\sigma^{2})\right).$$

**Figure 1:**
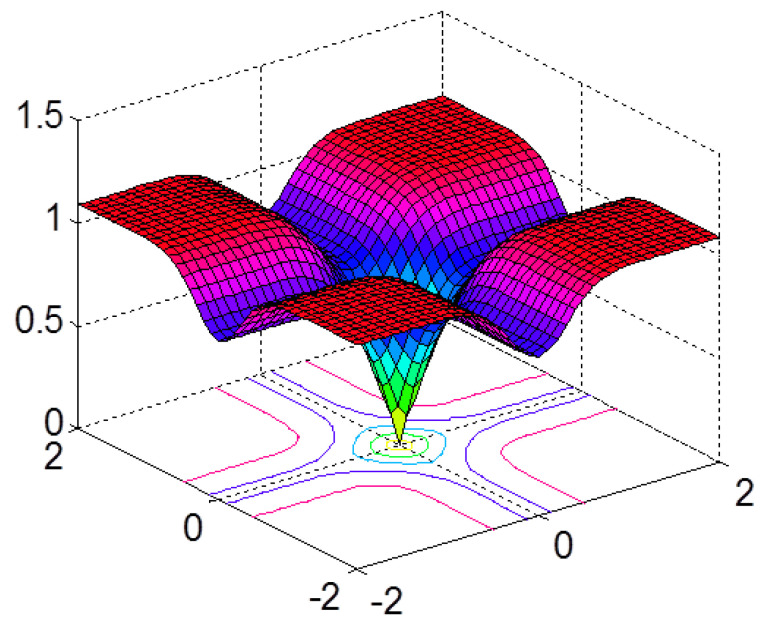
Correntropy-induced metric (CIM) surface plot showing distance regions (σ=0.02).

In Equation (12), if $|x_{i}|>\delta ,\forall x_{i}\neq 0$, then as σ→0, it can become close to the $\ell_{0}$ norm, where δ is a small positive number. Figure 1 shows the surface of the CIM(X,0) with $X={\left[x_{1},x_{2}\right]}^{T}$, which is plotted as a function of $x_{1}$ and $x_{2}$. Due to its relation with correntropy, this nonlinear metric is called the correntropy-induced metric (CIM) and can provide a good approximation for the $\ell_{0}$ norm. Hence, it favors sparsity and can be used as a sparse principal component (SPC) to exploit the system sparsity in SSI scenarios; a proof can be found elsewhere [5].

As an approximation of the $\ell_{0}$ norm, the CIM favors sparsity and can be used as a penalty term in the SSI problem. The CIMMCC algorithm has been proposed [22] to solve sparse channel estimation in an impulsive noise environment. The goal of this work is to develop a robust and sparse AFA combining the BCNMCC and CIM. The CIM constraint imposes a zero attraction of the filter coefficients according to the relative value of each coefficient among all the entries which, in turn, leads to improved performance when the system is sparse.

#### 3.1.2. CIM-BCNMCC

In general, the SPC-aware AFAs are designed by adding a derivative of an approximated $\ell_{0}$ norm with respect to the weight in the update equation of a given original AFA. Naturally, we can develop the BCNMCC algorithm with CIM (denoted as CIM-BCNMCC) by combining Equation (10) and the gradient of Equation (12) with a sparse controlling factor. Then, we obtain the weight update vector of the CIM-BCNMCC algorithm as

(13) $$\begin{array}{l} w(n+1)=\left(1+\mu \exp(\frac{v^{2}(n)}{-2\sigma^{2}})\frac{\sigma_{in}^{2}}{{\bar{u}}^{T}(n)\bar{u}(n)+\varepsilon }\right)w(n)\\ +\mu \exp(-\frac{{\bar{e}}^{2}(n)}{2\sigma^{2}})\frac{\bar{e}(n)\bar{u}(n)}{{\bar{u}}^{T}(n)\bar{u}(n)+\varepsilon }-\rho \frac{1}{M\sigma_{1}^{3}\sqrt{2\pi }}w(n)\exp(-\frac{w{\left(n\right)}^{2}}{2\sigma_{1}^{2}}) \end{array}$$

where ρ is a sparse controlling factor. It is worth noting that the kernel width $\sigma_{1}$ should be selected suitably to ensure that the CIM is closer to the $\ell_{0}$ norm.

**Remark** **1.** *The CIM-BCNMCC takes advantage of the BCNMCC algorithm and CIM; hence, it can solve the SSI problem under noisy input and output noise with impulsive characteristics. We can know that the CIM-BCNMCC will reduce to the BCNMCC algorithm when*ρ=0. *As a result, the extra computation complexity is from the third term in the right-hand side of Equation (13) compared with BCNMCC, but it can obtain a more perfect identification effect*.

### 3.2. BCPNMCC

#### 3.2.1. PMCC

The proportionate MCC algorithm has been proposed previously [27], and its weight update equation is

(14) $$w(n+1)=w(n)+\mu G(n)\exp(-\frac{e^{2}(n)}{2\sigma^{2}})e(n)u(n)$$

where $G(n)=diag(g_{1}(n),g_{2}(n),\ldots ,g_{M}(n))$ is a diagonal matrix which can update the step size of each tap adaptively. The individual gain $g_{l}(n)$ governing the step size adjustment of the *n*th weight coefficient is defined as

(15) $$g_{l}(n)=\frac{\gamma_{l}(n)}{\sum_{i=1}^{M}\gamma_{i}(n)}\;1\leq l\leq M$$

and

(16) $$\gamma_{l}(n)=\max(\xi \max(\delta ,|w_{1}(n)|,|w_{2}(n)|,\ldots ,|w_{M}(n)|),|w_{l}(n)|)$$

where ξ and δ are positive parameters, with typical values ξ=5/M, δ=0.01.

#### 3.2.2. BCPNMCC

In this subsection, we mainly develop a bias-compensated PNMCC algorithm to improve the performance of the PMCC algorithm for the noisy input case; its derivation will be given carefully. Just like the BCNMCC algorithm, we introduce a new term B(n) into Equation (14) to compensate for the bias caused by the input noise:

(17) $$w(n+1)=w(n)+\mu G(n)\exp(-\frac{{\bar{e}}^{2}(n)}{2\sigma^{2}})\frac{\bar{e}(n)\bar{u}(n)}{{\bar{u}}^{T}(n)G(n)\bar{u}(n)+\varepsilon }+B(n).$$

Considering the input noise, the output error is then denoted as

(18) $$\begin{array}{l} \bar{e}(n)=d(n)-{\bar{u}}^{T}(n)w(n)=d(n)-(u(n)+v_{in}^{T}(n)w(n))\\ =u^{T}(n)\tilde{w}(n)+v(n)-v_{in}^{T}(n)w(n)=e_{w}(n)+v(n)-v_{in}^{T}(n)w(n) \end{array}$$

where $e_{w}(n)=u^{T}(n)\tilde{w}(n)$ stands for the a priori error. Then, combining Equation (17) and WEV yields

(19) $$\tilde{w}(n+1)=\tilde{w}(n)-\mu G(n)\exp(-\frac{{\bar{e}}^{2}(n)}{2\sigma^{2}})\frac{\bar{e}(n)\bar{u}(n)}{{\bar{u}}^{T}(n)G(n)\bar{u}(n)+\varepsilon }-B(n).$$

In order to obtain B(n), taking the expectation on both sides of Equation (19) for the given $\bar{u}(n)$, we have

(20) $$E\left[\tilde{w}(n+1)|\bar{u}(n)\right]=E\left[\tilde{w}(n)|\bar{u}(n)\right]-\mu E\left[f(\bar{e}(n))\frac{G(n)\bar{e}(n)\bar{u}(n)}{{\bar{u}}^{T}(n)G(n)\bar{u}(n)+\varepsilon }|\bar{u}(n)\right]+E\left[B(n)|\bar{u}(n)\right].$$

By using the unbiasedness criterion given by Equation (8) in Equation (20), we obtain

(21) $$E\left[B(n)|\bar{u}(n)\right]=\mu E\left[f(\bar{e}(n))\frac{G(n)\bar{e}(n)\bar{u}(n)}{{\bar{u}}^{T}(n)G(n)\bar{u}(n)+\varepsilon }|\bar{u}(n)\right]$$

where $f(\bar{e}(n))$ denotes a nonlinear function of the estimation error defined by

(22) $$f(\bar{e}(n))=\exp(-\frac{{\bar{e}}^{2}(n)}{2\sigma^{2}})=\exp(-\frac{{\left(e_{w}(n)+v(n)-v_{in}{}^{T}(n)w(n)\right)}^{2}}{2\sigma^{2}}).$$

To derive the BCPNMCC algorithm reliably, the following commonly used assumptions [29,30,34] should be given firstly.

**Assumption** **1.** *The signals*v(n), $v_{in}(n)$, u(n)*, and*$\tilde{w}(n)$*are statistically independent*.

**Assumption** **2.** *The nonlinear function of the estimation error*f(v(n))*,*$v_{in}(n)$*,*G(n)*, and*$\bar{e}(n)$*are statistically independent*.

**Assumption** **3.** *The successive increments of tap weights are independent of one another, and the error and input vector sequences are statistically independent to one another*.

In order to facilitate the nonlinear function $f(\bar{e}(n))$ and simplify the mathematical derivation, we take the Taylor expansion of $f(\bar{e}(n))$ with respect to $e_{w}(n)-v_{in}^{T}(n)\tilde{w}(n)$ around v(n) and use Equation (22) to yield

(23) $$f(\bar{e}(n))\approx f(v(n))+f\prime (v(n))[e_{w}(n)-v_{in}{}^{T}(n)w(n)]+o\left[{\left[e_{w}(n)-v_{in}{}^{T}(n)w(n)\right]}^{2}\right].$$

Using Equation (23), we obtain the following approximation of Equation (21):

(24) $$\begin{array}{l} E\left[f(\bar{e}(n))\frac{G(n)\bar{e}(n)\bar{u}(n)}{{\bar{u}}^{T}(n)G(n)\bar{u}(n)+\varepsilon }|\bar{u}(n)\right]\approx E\left[f(v(n))\frac{G(n)\bar{e}(n)\bar{u}(i)}{{\bar{u}}^{T}(n)G(n)\bar{u}(n)+\varepsilon }|\bar{u}(n)\right]\\ +E\left[f\prime (v(n))[e_{w}(n)-v_{in}{}^{T}(n)w(n)]\frac{G(n)\bar{e}(n)\bar{u}(n)}{{\bar{u}}^{T}(n)G(n)\bar{u}(n)+\varepsilon }|\bar{u}(n)\right]\\ +E\left[o\left[{\left[e_{w}(n)-v_{in}{}^{T}(n)w(n)\right]}^{2}\right]\frac{G(n)\bar{e}(n)\bar{u}(n)}{{\bar{u}}^{T}(n)G(n)\bar{u}(n)+\varepsilon }|\bar{u}(n)\right] \end{array}.$$

In general, the a priori error $e_{w}(n)$ converges to a small value when the algorithm is close to its steady state, and it can be ignored with respect to the environmental noise when the step size is small [6]. Under Assumptions 1, 2, and 3 and considering the fact aforementioned, the second part of Equation (24) becomes

(25) $$\begin{array}{c} E\left[f\prime (v(n))[e_{w}(n)-v_{in}{}^{T}(n)w(n)]\frac{G(n)\bar{e}(n)\bar{u}(n)}{{\bar{u}}^{T}(n)G(n)\bar{u}(n)+\varepsilon }|\bar{u}(n)\right]\\ \approx -E\left[f\prime (v(n))v_{in}{}^{T}(n)w(n)\frac{G(n)\bar{e}(n)\bar{u}(n)}{{\bar{u}}^{T}(n)G(n)\bar{u}(n)+\varepsilon }|\bar{u}(n)\right]\approx 0 \end{array}.$$

Furthermore, the third part of Equation (24) becomes

(26) $$E\left[o\left[{\left[e_{w}(n)-v_{in}{}^{T}(n)w(n)\right]}^{2}\right]\frac{G(n)\bar{e}(n)\bar{u}(n)}{{\bar{u}}^{T}(n)G(n)\bar{u}(n)+\varepsilon }|\bar{u}(n)\right]\approx 0.$$

Combining Equations (24)–(26), and applying Assumption 3, we have

(27) $$\begin{array}{l} E\left[f(\bar{e}(n))\frac{G(n)\bar{e}(n)\bar{u}(n)}{{\bar{u}}^{T}(n)G(n)\bar{u}(n)+\varepsilon }|\bar{u}(n)\right]\\ \approx E\left[f(v(n))\frac{G(n)\bar{e}(n)\bar{u}(n)}{{\bar{u}}^{T}(n)G(n)\bar{u}(n)+\varepsilon }|\bar{u}(n)\right]=E[f(v(n)|\bar{u}(n))]E\left[\frac{G(n)\bar{e}(n)\bar{u}(n)}{{\bar{u}}^{T}(n)G(n)\bar{u}(n)+\varepsilon }|\bar{u}(n)\right] \end{array}.$$

Considering the fact that $\bar{e}(n)=e(n)-v_{in}^{T}(n)w(n)$, we have

(28) $$E\left[\frac{G(n)\bar{e}(n)\bar{u}(n)}{{\bar{u}}^{T}(n)G(n)\bar{u}(n)+\varepsilon }|\bar{u}(n)\right]=\frac{E[G(n)\bar{e}(n)\bar{u}(n)|\bar{u}(n)]}{{\bar{u}}^{T}(n)G(n)\bar{u}(n)+\varepsilon }=\frac{E[G(n)|\bar{u}(n)]E[\bar{e}(n)\bar{u}(n)|\bar{u}(n)]}{{\bar{u}}^{T}(n)G(n)\bar{u}(n)+\varepsilon }$$

where

(29) $$\begin{array}{l} E\left[\bar{e}(n)\bar{u}(n)|\bar{u}(n)\right]=E\left[[e(n)+v_{in}^{T}(n)w(n)][u(n)+v(n)]|\bar{u}(n)\right]\\ =E\left[e(n)\bar{u}(n)|\bar{u}(n)\right]+E\left[e(n)v(n)|\bar{u}(n)\right]+E\left[v_{in}^{T}(n)w(n)u(n)|\bar{u}(n)\right]+E\left[v_{in}^{T}(n)w(n)v_{in}(n)|\bar{u}(n)\right] \end{array}.$$

Under Assumptions (1)–(3), we have

(30) $$\begin{array}{l} E\left[e(n)\bar{u}(n)|\bar{u}(n)\right]=E\left[[v(n)+u^{T}(n)\tilde{w}(n)][u(n)+v_{in}(n)]|\bar{u}(n)\right]\\ =E\left[v(n)u(n)|\bar{u}(n)\right]+E\left[v(n)v_{in}(n)|\bar{u}(n)\right]+E\left[u^{T}(n)\tilde{w}(n)u(n)|\bar{u}(n)\right]+E\left[u^{T}(n)\tilde{w}(n)v_{in}(n)|\bar{u}(n)\right]\\ =0 \end{array}$$

and

(31) $$E[v_{in}{}^{T}(n)w(n)u(n)|\bar{u}(n)]=0$$

(32) $$E[v_{in}{}^{T}(n)w(n)v_{in}(n)|\bar{u}(n)]=\sigma_{in}^{2}E[w(n)|\bar{u}(n)].$$

Combining Equations (28) and (29)–(32), we get

(33) $$E\left[\frac{\bar{e}(n)\bar{u}(n)}{{\bar{u}}^{T}(n)G(n)\bar{u}(n)+\varepsilon }|\bar{u}(n)\right]=E\left[\frac{\sigma_{in}^{2}w(n)}{{\bar{u}}^{T}(n)G(n)\bar{u}(n)+\varepsilon }|\bar{u}(n)\right].$$

Then, substituting Equations (28) and (33) into Equation (21) yields

(34) $$E\left[B(n)|\bar{u}(n)\right]=\mu E\left[\exp(\frac{v^{2}(n)}{2\sigma^{2}})|\bar{u}(n)\right]E\left[G(n)|\bar{u}(n)\right]E\left[\frac{\sigma_{in}^{2}w(n)}{{\bar{u}}^{T}(n)G(n)\bar{u}(n)+\varepsilon }|\bar{u}(n)\right].$$

Now, using the stochastic approximation, we have

(35) $$B(n)=\mu \exp(-\frac{v^{2}(n)}{2\sigma^{2}})\frac{G(n)\sigma_{in}^{2}}{{\bar{u}}^{T}(n)G(n)\bar{u}(n)+\varepsilon }.$$

Substituting Equation (35) into Equation (17), we obtain

(36) $$w(n+1)=\left(1+\mu \exp(-\frac{v^{2}(n)}{2\sigma^{2}})\frac{G(n)\sigma_{in}^{2}}{{\bar{u}}^{T}(n)G(n)\bar{u}(n)+\varepsilon }\right)w(n)+\mu \exp(-\frac{{\bar{e}}^{2}(n)}{2\sigma^{2}})\frac{G(n)\bar{e}(n)\bar{u}(n)}{{\bar{u}}^{T}(n)G(n)\bar{u}(n)+\varepsilon }.$$

As a result, the update equation of the proposed BCPNMCC algorithm is derived in Equation (36).

**Remark** **2.** *The structure of Equation (36) is similar to that of Equation (10), and the proposed BCPNMCC algorithm will reduce to the BCNMCC algorithm when the gain matrix is the unity matrix. In addition, we know that Equation (36) will be equivalent to Equation (15) when the input noise variance is zero. Hence, the proposed BCPNMCC algorithm shows advantages of both the BCNMCC and PMCC algorithms. Furthermore, the variance of the input noise is usually unknown and it should be estimated effectively; we employ the method proposed in [35] to estimate*$\sigma_{in}^{2}$*in this work*.

## 4. Simulation Results

**Figure 2:**
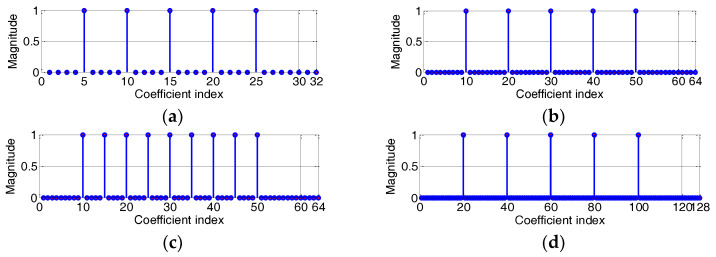
Impulsive response of the sparse system: (**a**) sparsity rate (SR) = 5/32; (**b**) SR = 5/64; (**c**) SR = 9/64; (**d**) SR = 5/128.

In this section, we present computer simulations to evaluate the performance of the proposed CIM-BCNMCC and BCPNMCC algorithms, and we select the MCC, CIM-MCC, and BCNMCC algorithms as comparison objects. In the following simulations, we set the parameter vector of an unknown time-varying system as shown in Figure 2.

In Figure 2, the memory size *M* is set at 32, 64, or 128 during different iterations. Here, we define the sparsity rate (SR) as

(37) $$SR=\frac{N_{non-zeros}}{M}$$

where $N_{non-zero}$ is the number of nonzero taps in $w^{o}$. We assume that the background noise v(n) has an α-stable distribution and that the input noise $v_{in}(n)$ is zero-mean white Gaussian noise. The characteristic function of the α-stable distribution is defined as

(38) $$f(t)=\exp\left\{j\theta t-\gamma {\left|t\right|}^{\alpha }\left[1+j\beta \mathrm{sgn}(t)S(t,\alpha )\right]\right\}$$

in which

(39) $$S(t,\alpha )=\{\begin{array}{c} \tan\frac{\alpha \pi }{2}\\ \frac{2}{\pi }\log|t| \end{array}\begin{array}{l} \begin{array}{cc} if & \alpha \neq 1 \end{array}\\ \begin{array}{cc} if & \alpha =1 \end{array} \end{array}$$

where α∈(0,2] is the characteristic factor, −∞<θ<+∞ is the location parameter, β∈[−1,1] is the symmetry parameter, and γ>0 is the dispersion parameter. We define the parameter vector of the characteristic function as $V_{\alpha -stable}(\alpha ,\beta ,\gamma ,\theta )$.

All results in the following simulations are obtained by averaging over 100 independent Monte Carlo runs to obtain the mean square deviation (MSD), defined as

(40) $$MSD=10\log_{10}\left(E\left(\frac{||w^{o}-w(n)|{|}^{2}}{||w^{o}|{|}^{2}}\right)\right).$$

In order to verify the performance of the new algorithms, we choose the optimal weight vector for different sparsities. The other parameters are set to achieve the best performance for each algorithm.

The kernel width σ of MCC is 20, and $\sigma_{1}$ is 0.02 for CIM; the input noise signal has a mean of 1 and variance $\sigma_{in}^{2}$ of 1, and the output measurement noise is set as $V_{\alpha -stable}(1.2,0,0.4,0)$. The positive parameter ρ is $1\;\times {\;10}^{-5}$, and ε=0.001.

### 4.1. Sparse System Identification

**Figure 3:**
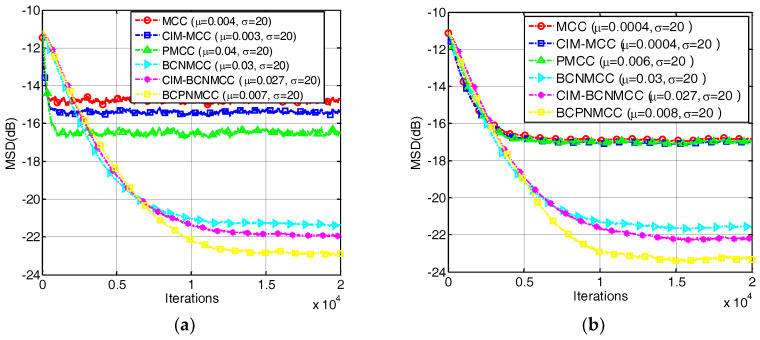
Convergence curves in terms of mean square deviation (MSD). The length of the filter is 64. SR = 5/64, $\sigma_{in}^{2}=1$, $\rho =1\;\times {\;10}^{-5}$, $\sigma_{1}=0.02$. (**a**) SR = 5/64; (**b**) SR = 9/64.

**Figure 4:**
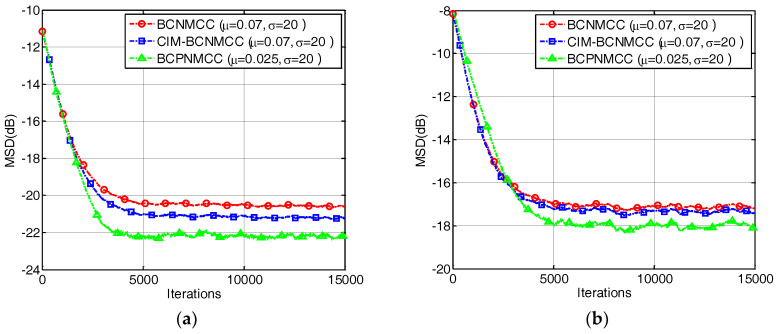
The convergence curves of the bias-compensated aware algorithms. (**a**) SR = 5/64; (**b**) SR = 9/64.

In the first example, we investigate the convergence performance of all algorithms mentioned above in terms of MSD under different sparsity rates. The step sizes are selected so that the same initial convergence speeds are obtained for all algorithms. The convergence curves are given in Figure 3a,b under different SRs (5/64 and 9/64). The results indicate that (1) the bias-compensated aware AFAs can achieve lower steady-state misadjustment than other algorithms because the bias caused by the noisy input can be effectively compensated by the bias compensation term; and (2) the noisy input has a remarkable influence on the identification performance of the AFAs for SSI. Consequently, it is meaningful to study the sparse-aware bias-compensated AFAs. In addition, we give the convergence curves of the BCNMCC, CIM-BCNMCC, and BCPNMCC algorithms with sparsity levels $N_{non-zeros}=5$ and $N_{non-zeros}=9$ in Figure 4. One can see that the proposed algorithms show better steady-state accuracy than the BCNMCC algorithm, which illustrates the advantages of the proposed methods for SSI. In particular, no matter how sparse it is, the BCPNMCC algorithm performs better than the CIM-BCNMCC algorithm, which is consistent with the results in [24]. Hence, we mainly perform simulations to investigate the performance of the proposed BCPNMCC algorithm in the following examples.

**Figure 5:**
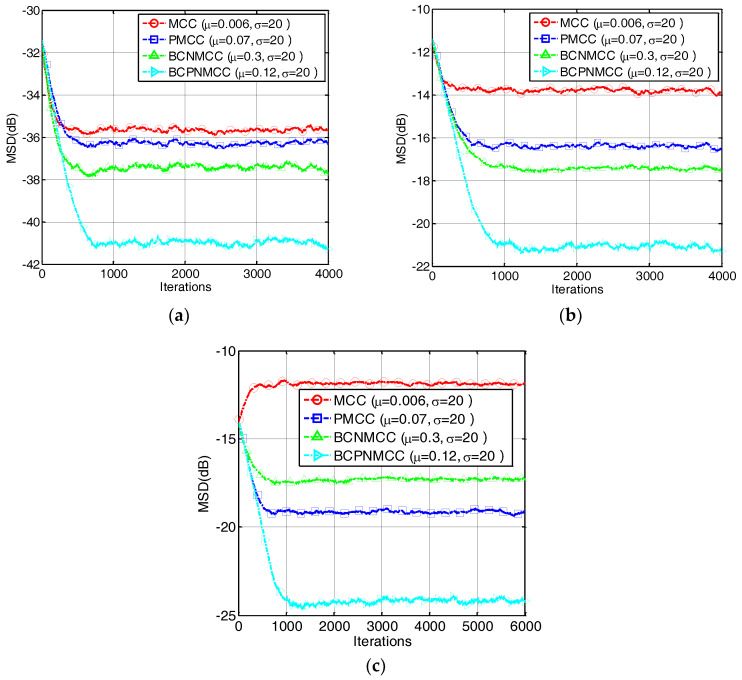
Convergence curves of four algorithms with different memory sizes. (**a**) SR = 5/32; (**b**) SR = 5/64; (**c**) SR = 5/128.

**Figure 6:**
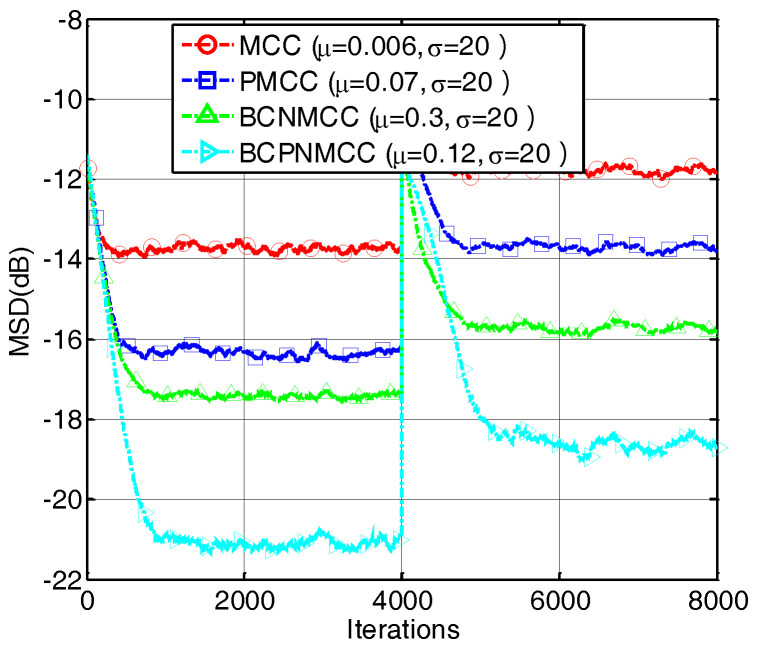
Convergence curves of four algorithms under different SRs.

In the second example, we present the convergence performance of the proposed and conventional algorithms obtained with different memory sizes represented by SR (a, SR = 5/32; b, SR = 5/64; and c, SR = 5/128). The results are shown in Figure 5; we conclude that the proportionate-type algorithms show the best performance, regardless of the length of the weight vector, compared with other algorithms. In addition, Figure 6 gives the results of a time-varying system identification case represented by SR, i.e., we set SR = 5/64 and SR = 9/64 in the first and second stages, respectively, to exhibit the tracking ability of the proposed algorithm. One can observe that the proposed algorithm can obtain the best performance no matter how sparse it is.

**Figure 7:**
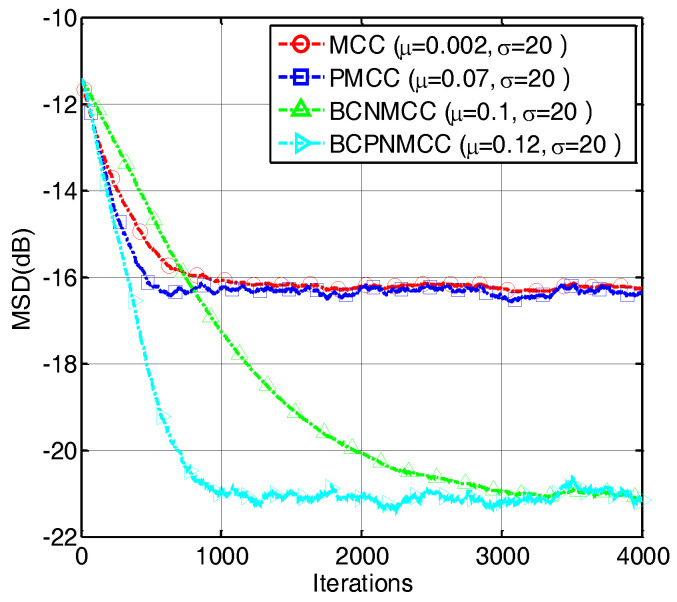
Convergence curves of four algorithms (SR = 5/64).

In the third example, we compare the convergence speed of the proposed algorithm with those of other algorithms. The step sizes are selected to obtain the same MSD for each algorithm. The convergence curves are shown in Figure 7; one can observe that the proposed BCPNMCC algorithm shows outstanding convergence speed compared with BCNMCC algorithm because of the adaptive step size adjustments by the gain matrix. Compared with the MCC algorithm, the BCPNMCC algorithm shows a better steady-state MSD because of the proportionate update scheme and bias compensating term.

**Figure 8:**
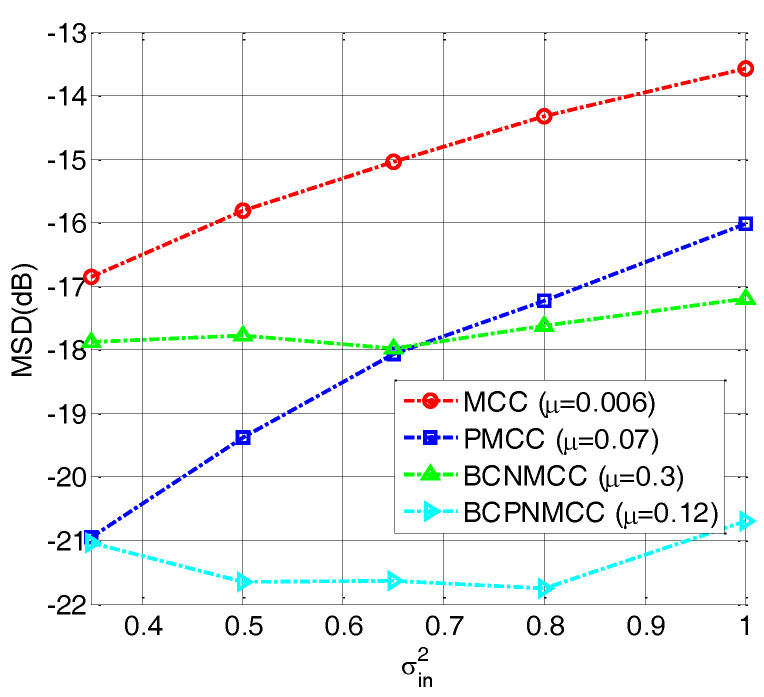
Steady-state MSDs with different values of $\sigma_{in}^{2}$; *σ* = 20, SR = 5/64.

**Figure 9:**
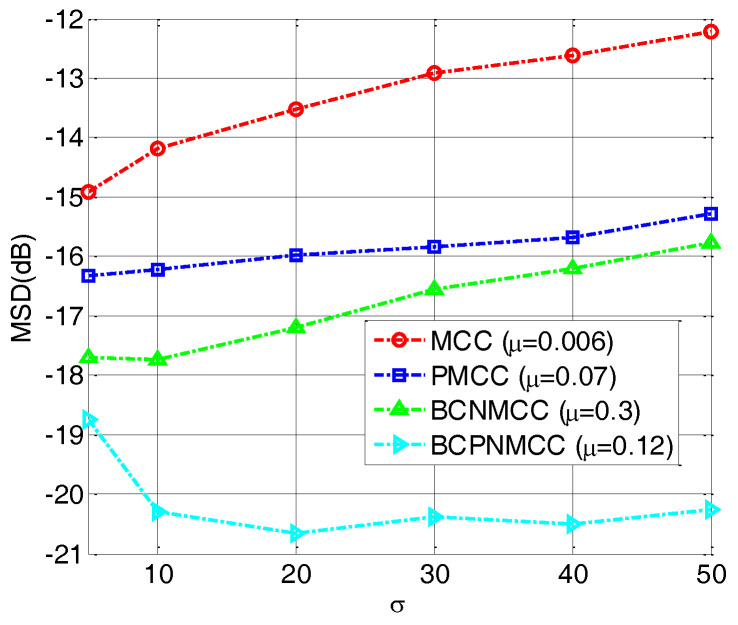
Steady-state MSDs with different values of *σ*; $\sigma_{in}^{2}=1$, SR = 5/64.

In the fourth example, we perform a simulation to further evaluate the robustness of the proposed algorithm with different values of $\sigma_{in}^{2}$ (0.3, 0.5, 0.65, 0.8, and 1). The other parameters are set the same as for the third example. The steady-state MSD is illustrated in Figure 8; we see that no matter how big $\sigma_{in}^{2}$ is, the bias-compensated aware algorithms (including BCNMCC and BCPNMCC) show better performance than their original versions (i.e., MCC and PMCC). Furthermore, the PMCC and BCPNMCC algorithms show higher steady-state accuracy than do the MCC and BCNMCC algorithms. Furthermore, the effect of the kernel width for the proposed algorithm is examined. We set σ at 5, 10, 20, 30, 40, and 50, respectively. Figure 9 gives the steady-state MSD result of each σ. It is obvious that (1) the proposed BCPNMCC algorithm outperforms the MCC, PMCC, and BCNMCC algorithms; and (2) the BCPNMCC algorithm achieves optimal performance when σ is 20, and a linear relationship does not exist between the performance and the value of σ, which means that the kernel width should be selected in different applications to achieve the desired performance.

**Figure 10:**
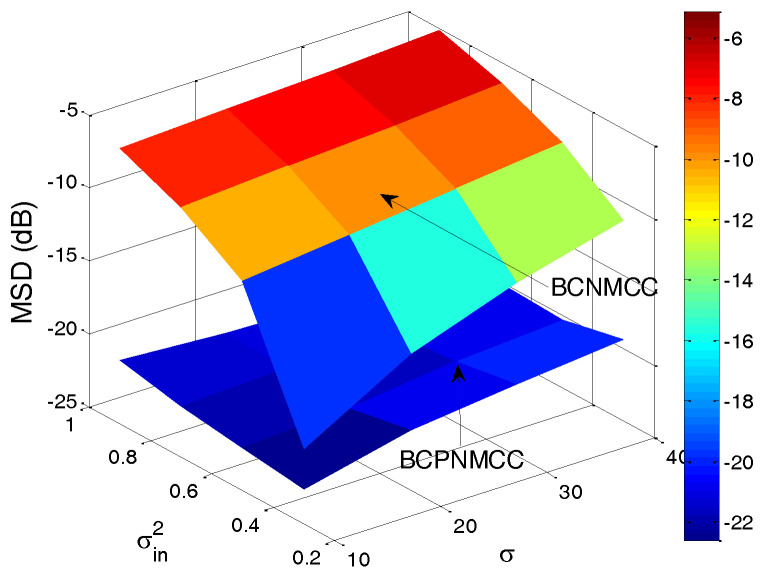
Steady-state MSDs of BCPNMCC for variations in σ and $\sigma_{in}^{2}$; SR = 5/64.

**Figure 11:**
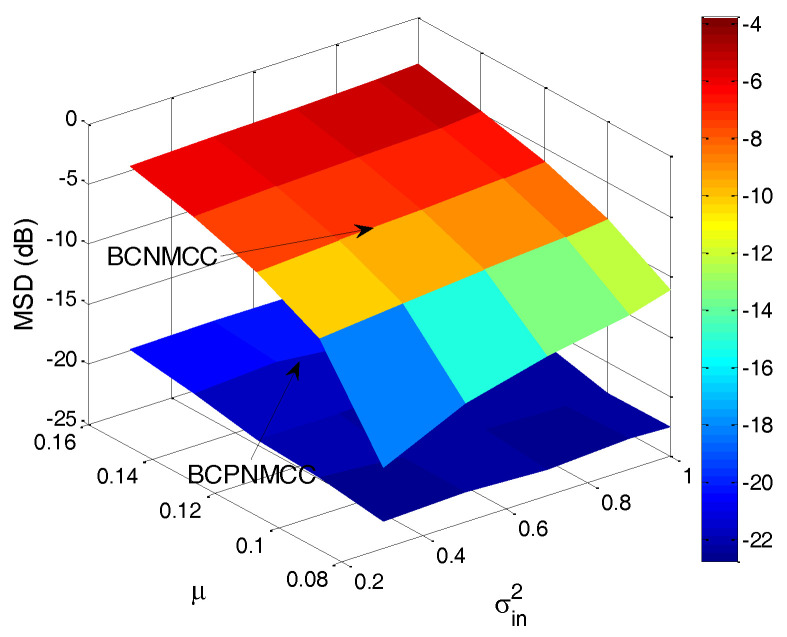
Steady-state MSDs of BCPNMCC for variations in μ and $\sigma_{in}^{2}$; SR = 5/64.

Finally, the joint effect of the performance on the free parameters is quantitatively investigated, with a curved surface for the relationship of steady-state MSD and parameter pairs (σ and $\sigma_{in}^{2}$, μ and $\sigma_{in}^{2}$) presented. The steady-state MSD results are given in Figure 10 and Figure 11. One can observe in Figure 10 that the steady-state accuracy will decrease with the input noise’s variance increasing; this result is most pronounced when the input noise’s variance and output noise’s variance are in the ranges of (0.2–1) and (10–40). A similar conclusion can be obtained in Figure 11: as the step size increases, the steady-state accuracy of the proposed algorithm decreases obviously, and it is most pronounced when the step size’s and input noise’s variance are in the ranges of (0.08–0.16) and (0.2–0.1). In addition, one can also see that the proposed algorithm outperforms the BCNMCC algorithm in this case.

### 4.2. Sparse Echo Channel Estimation

**Figure 12:**
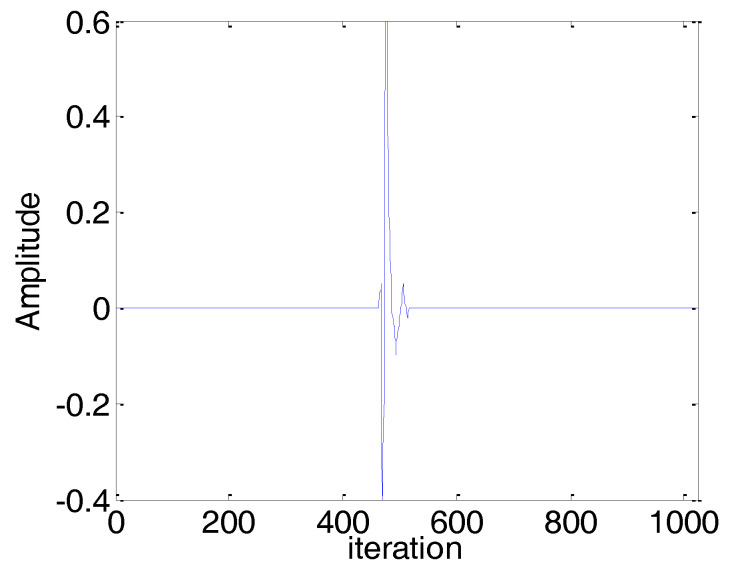
Impulse response of a sparse echo channel with length *M* = 1024 and 52 nonzero coefficients.

**Figure 13:**
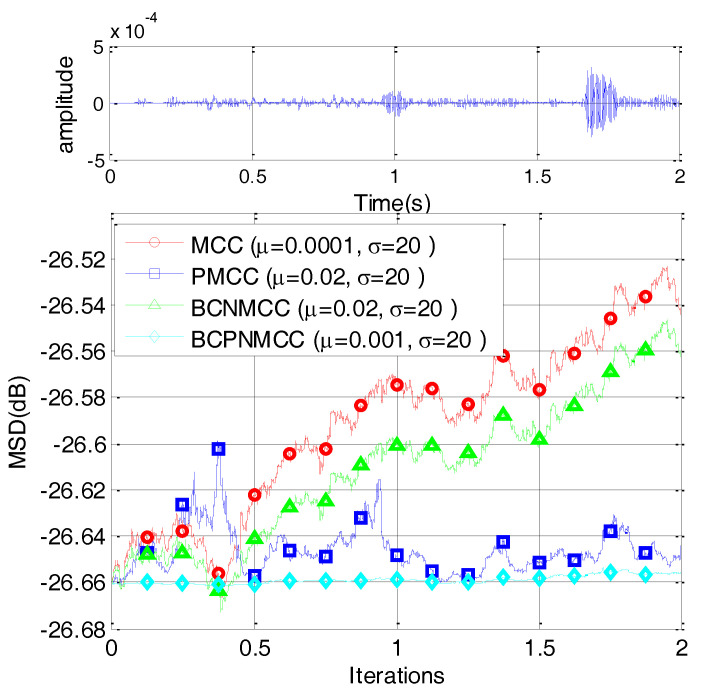
Convergence curves for sparse echo response with speech input.

In this subsection, we consider a computer simulation of the scenario of sparse echo channel estimation to evaluate the performance of the proposed BCPNMCC. The echo channel path is given in Figure 12, and it commutes to a channel with length *M* = 1024 only and 56 nonzero coefficients. We use a fragment of 2 s of real speech as the input signal, sampled at 8 kHz. The measurement impulsive noise is $V_{\alpha -stable}(1.2,0,0.2,0)$, and the input noise variance is $\sigma_{in}^{2}=0.25$. The other parameters are set in order to obtain the optimal performance for all algorithms. The convergence curves are shown in Figure 13. Compared with other algorithms, the proposed BCPNMCC algorithm works well in this practical scenario, again demonstrating the practical character of the proposed algorithm for engineering applications.

## 5. Conclusions

Sparse-aware BCNMCC algorithms for sparse system identification under noisy input and output noise with non-Gaussian characteristics have been developed in this paper, namely, the CIM-BCNMCC and BCPNMCC algorithms. They can achieve a higher identification accuracy of system parameters due to the introduction of the CIM penalty and the proportionate update scheme. In particular, the proposed BCPNMCC algorithm can also provide a faster convergence speed than the BCNMCC algorithm because of the adaptive step size adjustment by the gain matrix. More importantly, the BCPNMCC algorithm can lead to considerable improvements in the noisy input case for the SSI problem because it takes advantage of the bias-compensated term derived by the unbiasedness criterion. The simulation results demonstrate that (1) the CIM-BCNMCC and BCPNMCC algorithms perform better than other conventional algorithms; (2) the BCPNMCC algorithm outperforms CIM-BCNMCC when they are used for sparse system identification; and (3) no matter the step size, input noise’s variance, and kernel width, the BCPNMCC algorithm can always achieve the best performance.

## References

[1] Kalouptsidis N., Mileounis G., Babadi B. (2011). Adaptive algorithms for sparse system identification. Signal Process..

[2] Slock D.T.M. (1993). On the convergence behavior of the LMS and the normalized LMS algorithms. IEEE Trans. Signal Process..

[3] Walach E., Widrow B. (1984). The least mean fourth (LMF) adaptive algorithm and its family. IEEE Trans. Inf. Theory.

[4] Zerguine A. (2007). Convergence and steady-state analysis of the normalized least mean fourth algorithm. Digit. Signal Process..

[5] Liu W., Pokharel P., Principe J.C. (2007). Correntropy: Properties and applications in non-Gaussian signal processing. IEEE Trans. Signal Process..

[6] Chen B., Xing L., Liang J., Zheng N., Principe J.C. (2014). Steady-state mean-square error analysis for adaptive filtering under the maximum correntropy criterion. IEEE Signal Process. Lett..

[7] Chen B., Principe J.C. (2012). Maximum correntropy estimation is a smoothed MAP estimation. IEEE Signal Process. Lett..

[8] Chen B., Wang J., Zhao H., Zheng N., Principe J.C. (2015). Convergence of a fixed-point algorithm under maximum correntropy criterion. IEEE Signal Process. Lett..

[9] Chambers J., Avlonitis A. (1997). A robust mixed-norm adaptive filter algorithm. IEEE Signal Process. Lett..

[10] Breidt F.J., Davis R.A., Trindade A.A. (2001). Least absolute deviation estimation for all-pass time series models. Ann. Stat..

[11] Ma W., Chen B., Duan J., Zhao H. (2016). Diffusion maximum correntropy criterion algorithms for robust distributed estimation. Digit. Signal Process..

[12] Wu Z., Shi J., Zhang X., Chen B. (2015). Kernel recursive maximum correntropy. Signal Process..

[13] Chen B., Xing L., Zhao H., Zheng N., Principe J.C. (2016). Generalized correntropy for robust adaptive filtering. IEEE Trans. Signal Process..

[14] Wang Y., Li Y., Albu F., Yang R. (2017). Group-constrained maximum correntropy criterion algorithms for estimating sparse mix-noised channels. Entropy.

[15] Wu Z., Peng S., Chen B., Zhao H. (2015). Robust Hammerstein adaptive filtering under maximum correntropy criterion. Entropy.

[16] Li Y., Wang Y., Yang R., Albu F. (2017). A soft parameter function penalized normalized maximum correntropy criterion algorithm for sparse system identification. Entropy.

[17] Donoho D.L. (2006). Compressed sensing. IEEE Trans. Inf. Theory.

[18] Baraniuk R.G. (2007). Compressive sensing. IEEE Signal Process. Mag..

[19] Duttweiler D.L. (2000). Proportionate normalized least-mean-squares adaptation in echo cancelers. IEEE Trans. Speech Audio Process..

[20] Gu Y., Jin J., Mei S. (2009). *l*_0_ norm constraint LMS algorithm for Sparse system identification. IEEE Signal Process. Lett..

[21] Gui G., Adachi F. (2014). Sparse least mean fourth algorithm for adaptive channel estimation in low signal-to-noise ratio region. Int. J. Commun. Syst..

[22] Ma W., Qu H., Gui G., Xu L., Zhao J., Chen B. (2015). Maximum correntropy criterion based sparse adaptive filtering algorithms for robust channel estimation under non-Gaussian environments. J. Franklin Inst..

[23] Li Y., Wang Y., Albu F.A., Jiang J. (2017). General Zero Attraction Proportionate Normalized Maximum Correntropy Criterion Algorithm for Sparse System Identification. Symmetry.

[24] Li Y., Jin Z., Wang Y., Yang R. (2016). A robust sparse adaptive filtering algorithm with a correntropy induced metric constraint for broadband multipath channel estimation. Entropy.

[25] Ma W., Chen B., Qu H., Zhao J. (2016). Sparse least mean p-power algorithms for channel estimation in the presence of impulsive noise. Signal Image Video Process..

[26] Sayin M.O., Yilmaz Y., Demir A. (2015). The Krylov-proportionate normalized least mean fourth approach: Formulation and performance analysis. Signal Process..

[27] Ma W., Zheng D., Zhang Z. (2018). Robust proportionate adaptive filter based on maximum correntropy criterion for sparse system identification in impulsive noise environments. Signal Image Video Process..

[28] Jung S.M., Park P.G. (2013). Normalized least-mean-square algorithm for adaptive filtering of impulsive measurement noises and noisy inputs. Electron. Lett..

[29] Yoo J.W., Shin J.W., Park P.G. (2015). An improved NLMS algorithm in sparse systems against noisy input signals. IEEE Trans. Circuits Syst. II Express Briefs.

[30] Ma W., Zheng D., Tong X., Zhang Z., Chen B. (2017). Proportionate NLMS with Unbiasedness Criterion for Sparse System Identification in the Presence of Input and Output Noises. IEEE Trans. Circuits Syst. II Express Briefs.

[31] Zheng Z., Liu Z., Zhao H. (2017). Bias-compensated normalized least-mean fourth algorithm for noisy input. Circuits Syst. Signal Process..

[32] Zhao H., Zheng Z. (2016). Bias-compensated affine-projection-like algorithms with noisy input. Electron. Lett..

[33] Ma W., Zheng D., Li Y., Zhang Z., Chen B. (2017). Bias-compensated normalized maximum correntropy criterion algorithm for system identification with noisy Input. arXiv.

[34] Shin H.C., Sayed A.H., Song W.J. (2004). Variable step-size NLMS and affine projection algorithms. IEEE. Signal Process. Lett..

[35] Jo S.E., Kim S.W. (2015). Consistent normalized least mean square filtering with noisy data matrix. IEEE Trans. Signal Process..

